# MolClustPy: a Python package to characterize multivalent biomolecular clusters

**DOI:** 10.1093/bioinformatics/btad385

**Published:** 2023-06-16

**Authors:** Aniruddha Chattaraj, Indivar Nalagandla, Leslie M Loew, Michael L Blinov

**Affiliations:** R. D. Berlin Center for Cell Analysis and Modeling, University of Connecticut School of Medicine, Farmington, CT 06030, United States; R. D. Berlin Center for Cell Analysis and Modeling, University of Connecticut School of Medicine, Farmington, CT 06030, United States; R. D. Berlin Center for Cell Analysis and Modeling, University of Connecticut School of Medicine, Farmington, CT 06030, United States; R. D. Berlin Center for Cell Analysis and Modeling, University of Connecticut School of Medicine, Farmington, CT 06030, United States

## Abstract

**Summary:**

Low-affinity interactions among multivalent biomolecules may lead to the formation of molecular complexes that undergo phase transitions to become supply-limited large clusters. In stochastic simulations, such clusters display a wide range of sizes and compositions. We have developed a Python package, MolClustPy, which performs multiple stochastic simulation runs using NFsim (Network-Free stochastic simulator); MolClustPy characterizes and visualizes the distribution of cluster sizes, molecular composition, and bonds across molecular clusters. The statistical analysis offered by MolClustPy is readily applicable to other stochastic simulation software, such as SpringSaLaD and ReaDDy.

**Availability and implementation:**

The software is implemented in Python. A detailed Jupyter notebook is provided to enable convenient running. Code, user guide, and examples are freely available at https://molclustpy.github.io/

## 1 Introduction

Clustering of weakly interacting multivalent proteins and nucleic acids leads to biomolecular condensate formation via phase transition (Banani *et al.* 2017, Choi *et al.* 2020). These condensates are membrane-less sub-cellular compartments that play an important role in spatiotemporal regulation of cellular biochemistry (Lyon *et al.* 2021). Dysregulation of condensate biology is implicated in a series of pathological conditions (Alberti and Dormann 2019, Mathieu *et al.* 2020, Wang *et al.* 2021).

Since clustering of multivalent biomolecules underlies the condensate formation, it is important to model and characterize cluster formation. For example, size and composition of the condensates have important consequences in cell signaling (Su *et al.* 2016, Case *et al.* 2019). A better understanding of the biophysical properties of such condensates may facilitate testing hypotheses, interpreting experimental observations, and developing strategies to modulate such systems in a controlled manner, potentially leading to the identification of drug targets.

**Figure 1:**
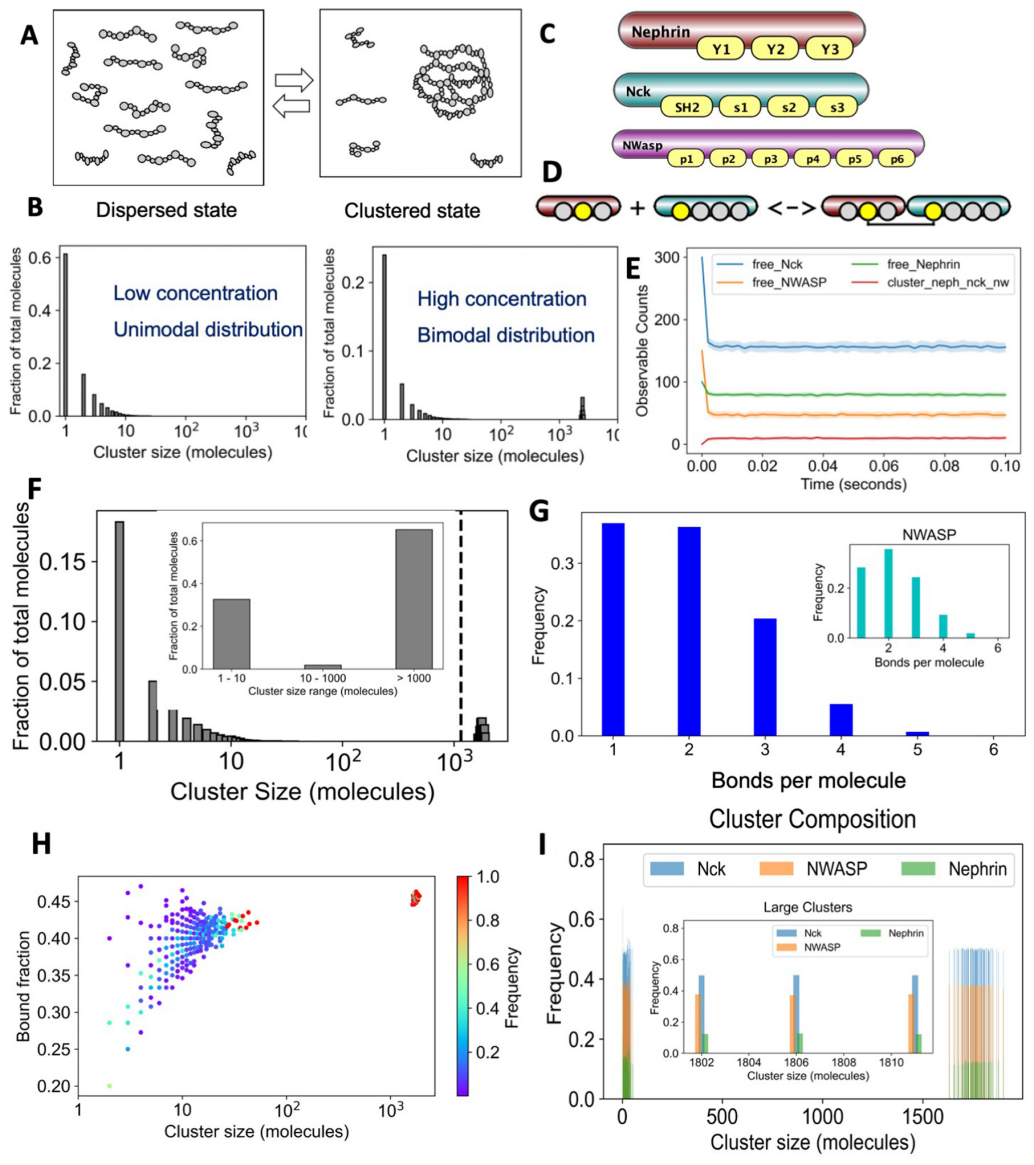
Characterization of molecular clusters. (A) Two states of molecular clusters: dispersed (monomeric and small oligomeric molecular complexes) and clustered (large clusters along with small clusters). (B) Quantification of cluster size distributions, with dispersed state being unimodal and clustered state being bimodal. (C) Rule-based depiction of three multi-site molecules Nephrin (three phosphotyrosines), Nck (one SH2 and three SH3 domains), and NWASP (six PRM domains). (D) A rule of Nephrin-Nck binding. Sites in gray do not affect the outcome of the interaction, so they can be bound or unbound. (E) Simulation output for several observables, illustrating the envelope across multiple trials. (F) ACO, a fraction of molecules in clusters of a given size. In insert is binning—the fractions of molecules in clusters of a certain size range. (G) The average number of bonds per molecule—across all molecules and for NWASP in the insert. (H) The distribution of binding saturations across molecular clusters of different sizes. (I) Composition of clusters, i.e. the relative abundance of each molecular type across cluster sizes. The inset shows compositions of a few large clusters.

Molecular clustering shows a switch-like behavior (phase transition) in a concentration-dependent manner (Chattaraj *et al.* 2021). Below a threshold concentration, molecules remain in the monomeric and small oligomeric states (Fig. 1A, dispersed state) that will manifest as a single homogeneous phase. Upon crossing the threshold, the system tends to form large clusters (Fig. 1A, clustered state). The co-existence of large clusters along with small clusters results in a splitting of the system into two distinct phases (dense and dilute). The cluster size distribution would shift from an exponentially decaying unimodal distribution (Fig. 1B, left) to a bimodal (bifurcated) distribution (Fig. 1B, right). A similar theoretical model reproducing these distributions was developed earlier (Goldman *et al.* 2004).

Weak molecular interactions result in a distribution of size and compositions of multi-molecular clusters (Mayer *et al.* 2009). Modeling this process requires multiple stochastic simulations and subsequent statistical analysis. We have previously used *ad hoc* code to analyze multivalent clustering for different biological systems (Falkenberg *et al.* 2013, Falkenberg *et al.* 2017, Chattaraj *et al.* 2019, Chattaraj *et al.* 2021). In this work, we present a Python package—MolClustPy that generalizes these methods to statistically analyze and visualize molecular clusters from multiple stochastic trials.

The user needs to describe the molecules and their interactions in the rule-based BioNetGen Language (BNGL) format (Blinov *et al.* 2004, Faeder *et al.* 2009, Harris *et al.* 2016). Then, MolClustPy will utilize the existing Python wrapper of BioNetGen (pyBioNetGen) to simulate the cluster formation for a user-defined number of times using an agent-based Network-Free stochastic simulator (NFsim) (Sneddon *et al.* 2011). The collected statistics averaged over multiple runs is used to provide average time courses of certain user-defined quantities (‘observables’), as well as characterization of the final state of the system.

## 2 Characterization of cluster composition

### 2.1 Biological system specification and simulation

The interaction of a limited supply of multivalent monomers produces individual clusters with variable sizes, molecular compositions, and connectivity. Therefore, stochastic agent-based modeling is appropriate for providing a comprehensive understanding at the system level. The most convenient way to define agents that are multi-site molecules and their interactions is via a modeling technique called rule-based modeling (Faeder *et al.* 2009).

To demonstrate the utility of our package, we will consider an experimentally well-characterized multivalent system—Nephrin, Nck, and NWASP (Li *et al.* 2012, Banjade and Rosen 2014). Molecules are defined as objects with multiple binding sites (Fig. 1C), and the interactions between molecules are defined as interactions leading to formation or breaking a bond between specific binding sites. Such interactions are best described by rules that define the input and output of interaction depending on the initial state of all binding sites. The rule in Fig. 1D defines the binding of Nephrin Y2 site to Nck SH2 site (sites shown in yellow). This rule corresponds to a potentially infinite number of individual interactions among molecular clusters, because other sites (shown in gray) may be bound to other molecules not shown in the rule definition. However, all interactions corresponding to the single rule are parameterized by the same on- and off-rate constants. Note that rules are not limited to binary site-site interactions but can be extended to encode arbitrary levels of complexities for interaction among multivalent molecules; for example, the binding strength of one molecular site may depend on the binding status of other sites of the same molecule to capture allosteric or cooperativity effects.

To stochastically simulate rule-based systems with a potentially infinite number of species and interactions, we use NFsim (Sneddon *et al.* 2011). NFsim simulation outputs consist of two major parts—observables and final molecular species. Observables are predefined global properties of the biological system whose concentrations are reported as a time course during the simulation, for example, concentrations of free molecules of a certain type. The final molecular species encompass the set of molecular complexes that exist at the end of each simulation run.

### 2.2 MolClustPy outputs

MolClustPy analyzes both observables and molecular clusters across multiple simulation runs. Figure 1E illustrates plots of the time courses for observables. Importantly, we demonstrate both the mean trajectory and the standard deviation shown as a fluctuation envelope. To demonstrate the width of the distribution, we plot observables for a lower concentration of molecules.

Figure 1F illustrates the cluster size distribution: each bar corresponds to the fraction of total molecules in each cluster size. In other words, this is a probability distribution of finding molecules in a certain cluster size. For example, the probability of finding a molecule in a monomeric form is 17%. The mean of the distribution is called average cluster occupancy (ACO) as shown by the dashed line. For a large cluster size range, a binned histogram might be helpful, as shown in the inset. Here, we see that 33% of all molecules are in small clusters of sizes up to 10, while around 62% of molecules are in large clusters (>1000 molecules).

Figure 1G captures the molecular crosslinking—the average number of bonds per molecule. For our mixed-valent system, Nephrin can have 1–3 bonds, Nck can have 1–4 bonds, and NWASP may have 1–6 bonds. Inspecting bond distribution of individual molecular types (NWASP, inset) informs the user on the degree of bound saturation for a given affinity and stoichiometry.

Figure 1H demonstrates the degree of bond saturation across molecular clusters of different sizes. Bound fraction (BF) is the ratio of bound sites over total sites present in that cluster. The color bar shows the relative frequencies of a given configuration. From the BF pattern, we see that there is a greater variability in smaller clusters, while large clusters converge to a characteristic fixed BF.

Figure 1I demonstrates the molecular composition of the clusters, giving the relative fraction of each molecular type within a given cluster size. Note that within each bar, the sum of all fractions is 1. The large clusters seem to have identical compositions (inset), suggesting a stoichiometry-driven clustering process, which is consistent with recent experimental finding (Case *et al.* 2019).

## 3 Implementation

The package is implemented in Python. It requires the pyBioNetGen package to run. MultiRun_BNG.py processes the BNGL file that defines molecules, initial species, rules of interactions, and optional observables. The user must input three parameters related to simulation: duration of each simulation, number of output time points, and number of stochastic trials. MultiRun_BNG.py calls the pyBioNetGen package, which executes NFsim simulations the required number of times. NFsim_data_analyzer.py collects the observables and molecular clusters to perform various statistical analyses. DataViz_NFsim.py visualizes the statistical outputs.

To quantify the cluster property distribution, we collect the molecular species (clusters) from multiple trials and perform statistical analysis on the combined dataset. A “cluster” is a molecular network where each node is a multivalent molecule, and the bonds comprise the edges. We analyze the network topology and their relative abundances. For example, the degree of a node gives the number of bonds coming out of that particular molecule. BF is then computed as the ratio of bound sites over total sites present in a molecule.

## 4 Conclusions

We have demonstrated the use of MolClustPy to characterize key properties of molecular clusters derived from multiple stochastic NFsim simulations. A limitation of BioNetGen/NFsim is that it ignores spatial factors that may control cluster growth and composition. These include steric and crowding effects as well as the geometric organization of binding sites. A related spatial issue is the importance of disordered proteins and protein domains in the formation of biomolecular condensates. The SpringSaLaD (Michalski and Loew 2016) or ReaDDy (Hoffmann *et al.* 2019) simulators can address these issues (at the cost of much longer computational times). MolClustPy can readily be adapted to accommodate the outputs from these spatial stochastic simulators.
